# Unusual onset of thyroid associated orbitopathy during pregnancy: case report and review of literature

**DOI:** 10.1186/s12902-020-00663-9

**Published:** 2020-12-15

**Authors:** Janos K. Aranyosi, Tamas Deli, Annamaria Erdei, Geza Toth, Attila Jakab, Mariann Fodor, Endre V. Nagy, Bernadett Ujhelyi

**Affiliations:** 1 Department of Ophthalmology, Faculty of Medicine, University of Debrecen, Nagyerdei krt 98, Debrecen, H-4032 Hungary; 2 Department of Obstetrics and Gynecology, Faculty of Medicine, University of Debrecen, Debrecen, Hungary; 3 Division of Endocrinology Department of Internal Medicine, Faculty of Medicine, University of Debrecen, Debrecen, Hungary; 4 Department of Internal Medicine, Szent Lázár Hospital, Salgótarján, Hungary

**Keywords:** TAO, Graves’ disease, Pregnancy, Orbitopathy, Smoking, Case report

## Abstract

**Background:**

Thyroid associated orbitopathy (TAO) is the most common extrathyroidal complication of Graves’ disease. The disease course ranges from mild, where symptomatic therapy is sufficient, to severe, where high dose steroid administration or orbital decompression surgery is required. Women of their reproductive age are more likely to be affected. Although pregnancy is a state of enhanced immune tolerance, TAO may develop or worsen in 0.2–0.4% of pregnant women.

**Case presentation:**

We present the case of a 19-year-old woman who has developed hyperthyroidism and progressive TAO during the second trimester of her third pregnancy, which has improved postpartum. The possible mechanisms and the importance of follow up in pregnancy is discussed.

**Conclusions:**

Expectant mothers with Graves’ disease require follow up of eye signs throughout pregnancy, preferably in the setting of a thyroid-eye clinic.

## Background

Graves’ disease (GD) and Hashimoto’s thyroiditis (HT) are autoimmune disorders, that may affect around 2% of the population [1]. GD occurs more frequently in women, smokers, patients suffering from other autoimmune diseases and those with a positive family history for thyroid autoimmunity [2]. GD is the most common cause of hyperthyroidism in women of reproductive age. It occurs before and during pregnancy in 0.4–1% and 0.2–0.4% of the cases, respectively [3, 4]. According to a recent review by Chin et al. in approximately 40% of the cases GD is accompanied by thyroid associated orbitopathy (TAO) [5]. In 5%, deterioration of visual acuity may occur [6]. In mild cases local therapy such as artificial tears, ointments, sunglasses and higher pillows at night are sufficient. Severe cases require systemic immunosuppressive therapy, usually with high dose corticosteroids [7]. Additional options are retrobulbar irradiation and orbital wall decompression surgery [8]. During pregnancy the therapeutic options are limited.

The majority of autoimmune diseases tend to improve during gestation due to the state of enhanced immune tolerance. Thyroid autoimmune diseases, multiple sclerosis and rheumatoid arthritis show improvement during pregnancy [9, 10]. There are a few exceptions; SLE and type 1 diabetes deteriorate [11].

In GD, humoral immune response predominates. Stimulating antibodies against the TSH receptor (TRAb) result in the clinical manifestations of the disease, including hyperthyroidism, goiter, orbitopathy and dermopathy [12]. The development or exacerbation of GD in pregnancy is rare and spontaneous improvement in the second trimester is typical. This is accompanied by the decrease of stimulating TRAbs [13, 14]. Relapse is often observed between 4 and 8 months after delivery. In this period, the enhanced immune tolerance ceases, resulting in elevated levels of TRAbs [14].

Here we present a case of GD with TAO, which developed in the second trimester of pregnancy.

## Case presentation

A 19-year-old pregnant woman has been referred to our department during her 3rd pregnancy for GD and new-onset TAO. Hyperthyroidism was diagnosed by her primary care physician at week 20 of pregnancy. Her laboratory results were: TSH 0.01 mU/L, FT4: 78,4 pmol/L (reference range: 12–22 pmol/L), FT3: 37.4 pmol/L (reference range: 3.1–6.8 pmol/L). For unknown reasons, she showed up three months later, at week 33 of pregnancy, with untreated hyperthyroidism. She did not report any other disease. She has had no history of thyroid disease and no pre-pregnancy TSH values were available. The two previous pregnancies conceived naturally and were completed by cesarean section due to cephalopelvic disproportion. She smoked 20 cigarettes daily during pregnancy and failed to quit smoking in spite of repeated counseling.

On presentation at week 33 her main complaints were proptosis and limited movement of the right eye with accompanying pain. She recalled that her first symptom, the proptosis appeared around week 14 of gestation. On admission TSH and thyroid hormone levels were in the hyperthyroid range (Table 1). Her TRAb level was 2.4 U/L (reference range:< 1 U/L), while the anti-thyroglobulin antibody level was 1324.0 IU/ml (reference range: < 60 IU/ml.). In spite of current recommendations [15] an initial dose of metimazol 50 mg/day has been chosen; severe type T3 hyperthyriodism was considered to carry high risk of preterm delivery, while there was no fetal risk of thiamazole in the 3rd trimester. After 3 days, the dose of thiamazole was reduced to 30 mg per day. Examination showed marked exophthalmos on the right side with restricted and painful eye movement, decreased best corrected visual acuity (20/32) and eyelid edema. Proptosis was 28 and 21 mm at the right and left sides, respectively by Hertel exophthalmometry at 90 mm (regional normal < 20 mm). The disease was graded as severe on the EUGOGO scale [16]. Clinical Activity Score (CAS) was 6/7 and 0/7 on the right and left sides, respectively.

**Table 1:** Hormone levels and thiamazole doses

Gestational age	sTSH (mU/L)	fT4 (pmol/L)	fT3 (pmol/L)	Thiamazole (mg/day)
**Week 20**	0.01	78.4	37.4	–
**Week 33**	< 0.005	40.9	18.1	50
**Week 34**	not done	31.9	12.9	30
**Week 36**	< 0.005	29.1	11.3	30
**Week 38**	< 0.005	29.5	9.6	30
**Post partum day 6**	< 0.005	44.0	19.3	50

**Normal ranges:** sTSH: 0.3–4.2 mU/L; fT4: 12.0–22.0 pmol/L; fT3: 2.4–6.3 pmol/L

The fetus showed no signs of intrauterine growth restriction, no tachycardia or goiter and the growth rate was satisfactory on follow up according to the ultrasonography. From week 34, the therapeutic dose of thiamazole was reduced to two times 15 mg daily (Table 1). Eye examination on week 38 showed improvement in visual acuity, CAS and proptosis. (Table 2), although retrobulbar pain, pain on eye movement, eyelid edema and conjunctival redness remained. Because the patient did not develop sight-threatening TAO, the thyroid-eye clinic team decided not to initiate high-dose corticosteroid therapy.

**Table 2:** Ophthalmologic examinations

Gestational age	Hertel (mm) o.d. – o.s.	CAS o.d. - o.s.	Visual acuity o.d. - o.s.
**Week 33**	28–21/90	6–0	20/32–20/20
**Week 38**	26–18/90	5–0	20/25–20/20
**Post partum day 3**	26–18/90	4–0	20/20–20/20

**Fig. 1:**
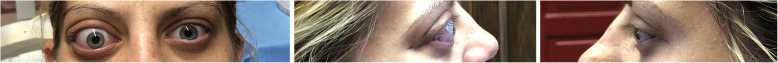
The patient’s eyes on day 3 postpartum. Exophthalmos on the right side was still present. Visual acuity returned to 20/20

A healthy female baby was delivered through cesarean section on week 39. Both TAO and cephalopelvic disproportion were contraindications of vaginal delivery. The newborn did not show clinical signs of thyroid dysfunction, and day 3 TSH was in the reference newborn range. The mother’s thyroid hormone levels started to rise after delivery; thiamazole dose was modified accordingly, supplemented with bisoprolol. Her eye complaints substantially improved the next day after the cesarean section, CAS declined, and visual acuity returned to normal (Table 2). Proptosis, eyelid and conjunctival redness and swelling of the eyelid and caruncula were still present on day 3 postpartum (Fig. 1). MRI examination was to be performed, however, the patient declined it. For the relapsing Graves’ disease and orbital involvement, total thyroidectomy was scheduled; later she declined and she and her baby were lost for follow up.

## Discussion and conclusions

Graves’ disease is caused by autoantibodies, which bind to the thyrotropin receptor on thyroid follicular cells and orbital fibroblasts, causing the overproduction of thyroid hormones [17] and in 40% of these patients TAO, respectively [6]. The autoimmune inflammation of the retrobulbar connective tissue is characterized by the presence of lymphocytes and macrophages and the overproduction of connective tissue matrix by fibroblasts [18, 19].

During normal pregnancy, several physiological changes occur [20]. Both red blood cell mass and plasma volume increase in pregnancy; as the latter increase is more marked, physiological haemodilution is present. Heart rate and stroke volume increase while vascular resistance falls. Glucose tolerance decreases, as does biliary excretion. Estrogen and progesterone levels rise dramatically, estrogen levels being 100-times higher than in the menstrual cycle [20]. The number of natural killer cells (NK) might be normal; however their cytolytic activity is decreased [21]. The number of circulating plasma cells and B-cells remains normal, as do antibody response and antibody production [22]. Although there is no significant change in the number of circulating T-lymphocytes during pregnancy, a slight decline may occur in the proportion of CD4+ cells resulting in suppressed immune activity [23]. Regulatory CD4+ CD25 + T cells, or Treg cells are responsible for regulating Th1-type and Th2-type activities. Th1-type and Th2-type immune processes are involved in cellular and humoral immunity, respectively [24]. In early pregnancy the number of Treg cells increases rapidly, reaching the peak in the second trimester [25]. They can suppress both Th1-type and Th2-type reactivity against fetal alloantigens. Th1 clones are more sensitive to the Treg effect, which leads to the predominance of Th2 clones over Th1 cellular immune response; the cytokine balance is driven away from the adverse effects of Th1 cell activity which could lead to abortion [26]. Therefore, the proper balance of Th1/Th2 cell activities has an important role in maintaining pregnancy. A clear decline in the number of Treg cells is observed in the weeks before delivery. This restoration of the non-pregnant or pre-pregnancy state may facilitate the exacerbation of autoimmune diseases after delivery [27, 28].

Pregnancy has a complex effect on the course of autoimmune disorders. The majority of autoimmune diseases tend to improve during pregnancy, except for SLE and type 1 diabetes, which usually worsen [9, 11]. Stable Graves’ disease on thiamazole may transiently worsen in the first trimester due to the thyroid stimulating effect of the hCG rise [29]. There is an improvement due to physiological immune tolerance in the second trimester. A relapse in the postpartum period, when the immune function returns to a normal, is common [30]. Therefore, improvement in the disease activity of both GD and TAO can be anticipated during pregnancy. While this is true for GD, unfortunately, less is known about the course of TAO [19, 31]; GD and TAO might follow separate courses during pregnancy. In one study, 5 out of 9 women with mild to moderate eye symptoms developed proptosis during pregnancy; on the other hand, 70% of patients with pre-existing disease had either improvement or no change in their eye signs and 30% experienced worsening in the postpartum period [32].

In the present case, a relapse of Graves’ disease was diagnosed during gestation. TAO appeared around the 14th week of pregnancy whereas she had no similar complaints during her two previous pregnancies. Her orbital condition was severe on presentation. It was not known if the reduced visual acuity was a new complaint. For this reason, both visual acuity and critical fusion frequency were tested on alternate days. We considered the orbitopathy moderate to severe [7], definitely not sight threatening; VA was 20/32. According to the EUGOGO guidelines [7], we decided to restore euthyroidism first and urged the patient to quit smoking. The latter measure is known to be nearly as effective as corticosteroids. As VA improved and signs reduced, there was no need to initiate immunosuppressive treatment.

We believe that the major contributors to TAO development and worsening during pregnancy have been the hypervolemia-related physiological changes and their reflections in the orbital tissue. A minor increase of orbital tissue volume can result in the worsening of TAO, as any volume excess in the orbital cone will lead to elevated cone pressure, further stretching of the rectus muscles, and decreased venous drainage into the cavernous sinus [33]. The usual rapid volume redistribution after delivery may explain the immediate improvement of TAO. It is not entirely clear why some of the TAO patients develop asymmetric disease; at presentation, other causes of unilateral exophthalmos have to be ruled out [34]. We [35] and others found that the orbital process is unequivocally two-sided by imaging, even in those cases where one side appears completely spared. MRI was planned but was postponed until after delivery in accordance with the practice guidelines collaboratively developed by the American College of Radiology (ACR) and the Society for Pediatric Radiology (SPR) for any trimester in pregnancy [36]. The patient failed to show up for imaging.

Further, the pregnant patient smoked 20 cigarettes daily. Although there were no data on the number of cigarettes smoked during her previous two pregnancies, the increasing amount of cigarettes per day throughout the years may account for the appearance of TAO. Furthermore, this case is unusual**,** because Graves’ hyperthyroidism did not limit itself during pregnancy; persisting thyroid dysfunction may have been a contributor to the worsening of TAO.

The limitation of our case report is that we could perform neither the MRI examination nor the follow-up visit due to low compliance of the patient. Her low socioeconomic status may account for both the disappearance from medical care, as well as smoking throughout pregnancy.

Both smoking and hypervolemia may have contributed to the development of TAO. The deterioration in the 3rd trimester may have most probably been volume-related as the eye signs rapidly regressed after delivery, while she maintained the same smoking intensity. Further improvement of the eye signs was confirmed by the patient at the single phone call she returned. Expectant mothers with Graves’ disease require follow up of eye signs throughout pregnancy, preferably in the setting of a thyroid-eye clinic.

## Data Availability

The datasets used and/or analysed during the current study are available from the corresponding author on reasonable request.

## Abbreviations

TAO: Thyroid associated orbitopathy
GD: Graves’ disease
HT: Hashimoto’s thyroiditis
TRAb: TSH receptor autoantibodies
CAS: Clinical Activity Score
NK: Natural killer cells
VA: Visual acuity
